# New horizons of biomaterials in treatment of nerve damage in diabetes mellitus: A translational prospective review

**DOI:** 10.3389/fendo.2022.1036220

**Published:** 2022-10-27

**Authors:** Alok Raghav, Manish Singh, Goo-Bo Jeong, Richa Giri, Saurabh Agarwal, Sanjay Kala

**Affiliations:** ^1^ Multidisciplinary Research Unit, Department of Health Research, Ganesh Shankar Vidyarthi Memorial (GSVM) Medical College, Kanpur, India; ^2^ Department of Neurosurgery, Ganesh Shankar Vidyarthi Memorial (GSVM) Medical College, Kanpur, India; ^3^ Department of Anatomy and Cell Biology, College of Medicine, Gachon University, Incheon, South Korea; ^4^ Kamlapat Singhania (KPS) Institute of Medicine, Ganesh Shankar Vidyarthi Memorial (GSVM) Medical College, Kanpur, India; ^5^ Department of Surgery, Ganesh Shankar Vidyarthi Memorial (GSVM) Medical College, Kanpur, India

**Keywords:** biomaterials, nerve damage, diabetes, diabetic neuropathy, cryogels, stem cells

## Abstract

**Background:**

Peripheral nerve injury is a serious concern that leads to loss of neuronal communication that impairs the quality of life and, in adverse conditions, causes permanent disability. The limited availability of autografts with associated demerits shifts the paradigm of researchers to use biomaterials as an alternative treatment approach to recover nerve damage.

**Purpose:**

The purpose of this study is to explore the role of biomaterials in translational treatment approaches in diabetic neuropathy.

**Study design:**

The present study is a prospective review study.

**Methods:**

Published literature on the role of biomaterials in therapeutics was searched for.

**Results:**

Biomaterials can be implemented with desired characteristics to overcome the problem of nerve regeneration. Biomaterials can be further exploited in the treatment of nerve damage especially associated with PDN. These can be modified, customized, and engineered as scaffolds with the potential of mimicking the extracellular matrix of nerve tissue along with axonal regeneration. Due to their beneficial biological deeds, they can expedite tissue repair and serve as carriers of cellular and pharmacological treatments. Therefore, the emerging research area of biomaterials-mediated treatment of nerve damage provides opportunities to explore them as translational biomedical treatment approaches.

**Conclusions:**

Pre-clinical and clinical trials in this direction are needed to establish the effective role of several biomaterials in the treatment of other human diseases.

## Introduction

**Figure 1:**
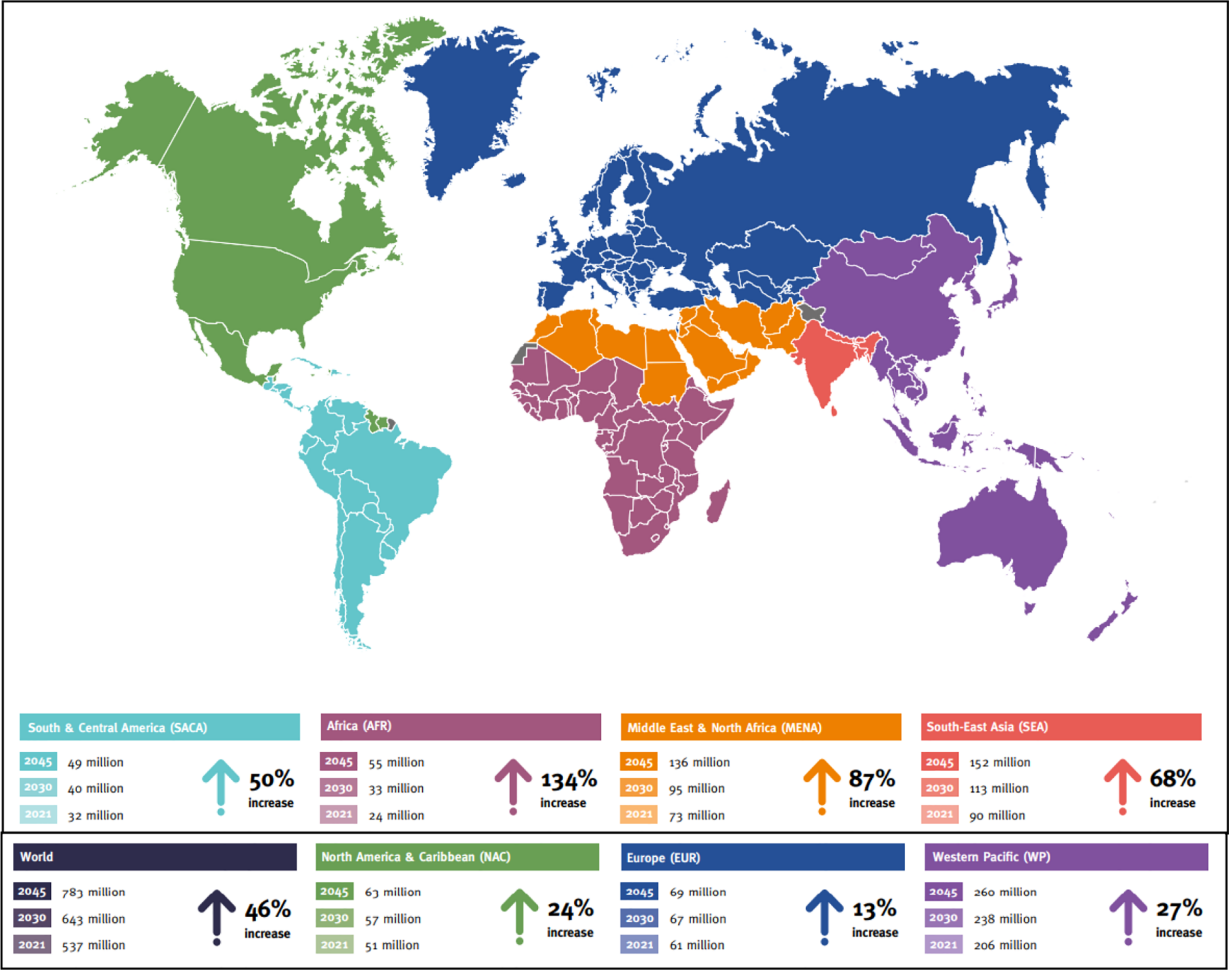
Projected number of people with diabetes worldwide and per IDF Region in 2021–2045 (20–79 years) (Source: International Diabetes Federation; 10^th^ Atlas).

Peripheral neuropathy (PN) is a highly complex and prevalent disease that involves the plexus of nerve tissues. In epidemiological studies from various regions in India, the overall prevalence of PN varied from 5 to 2400 per 10,000 population in various community studies (1). Neuropathy is characterized by pathologic cellular and non-cellular changes in the premises of the peripheral nerve, especially arising in type 1 diabetes (in which the pancreas does not produce insulin in sufficient quantity due to β-cell dysfunction) and type 2 diabetes mellitus (T2DM) (in which there is some insulin resistance) (2). In the United States and Europe, it is presumed that pre-diabetes is also the leading cause of peripheral neuropathy (3). In the United States, around 20 million people currently have neuropathic-related secondary complications and this number progressively doubles as the incidences of pre-diabetes and T2DM increase (4). Furthermore, the worldwide cases of pre-diabetes and diabetes are 316 million and 537 million, respectively, according to the International Diabetes Federation (IDF) (**Figure 1**) (4). Diabetes mellitus leads to several acute, chronic, diffuse, and focal neuropathy syndromes. Peripheral nervous system (PNS) damage, associated with diabetes includes bilateral and symmetric damage to feet nerves, having a distal-to-proximal gradient of severity also known as stocking-glove neuropathy (5). Peripheral diabetic neuropathy (PDN) has been defined as a “symmetrical, length-dependent sensorimotor polyneuropathy attributable to metabolic and micro-vessel alterations due to chronic hyperglycemia exposure and cardiovascular risk covariates” (6). PDN typically presents positive sensory symptoms in the feet that include pain, tingling, and prickling of sensations (paresthesias) along with negative symptoms such as numbness, loss of sensation (disordered sensory processing), episodes of pain in the feet region when touched (allodynia), and elevated sensitivity to noxious stimuli (hyperalgesia). The severity of PDN is associated with morbidity, lower limb fractures, ulceration, and amputations (7–10). **Table 1** shows the detailed classification of PDN. There is evidence of motor nerve dysfunction associated with distal toes weakness and, in vulnerable cases, the ankles and calves. It is a matter of debate as to why the sensory axons, compared to motor axons, are vulnerable to diabetes-associated complications.

**Table 1:** Classification of diabetic neuropathies.

A. Diffuse neuropathy
**DSPN**
•Primarily small-fiber neuropathy
•Primarily large-fiber neuropathy
•Mixed small- and large-fiber neuropathy (most common)
**Autonomic**
**Cardiovascular**
•Reduced HRV
•Resting tachycardia
•Orthostatic hypotension
•Sudden death (malignant arrhythmia)
**Gastrointestinal**
•Diabetic gastroparesis (gastropathy)
•Diabetic enteropathy (diarrhea)
•Colonic hypomotility (constipation)
**Urogenital**
•Diabetic cystopathy (neurogenic bladder)
•Erectile dysfunction
•Female sexual dysfunction
**Sudomotor dysfunction**
•Distal hypohydrosis/anhidrosis,
•Gustatory sweating
•Hypoglycemia unawareness
•Abnormal pupillary function
**B. Mononeuropathy (mononeuritis multiplex) (atypical forms)**
•Isolated cranial or peripheral nerve (e.g., CN III, ulnar, median, femoral, peroneal)
•Mononeuritis multiplex (if confluent may resemble polyneuropathy)
**C. Radiculopathy or polyradiculopathy (atypical forms)**
•Radiculoplexus neuropathy (a.k.a. lumbosacral polyradiculopathy, proximal motoramyotrophy)
•Thoracic radiculopathy
**Nondiabetic neuropathies common in diabetes**
•Pressure palsies
•Chronic inflammatory demyelinating polyneuropathy
•Radiculoplexus neuropathy
•Acute painful small-fiber neuropathies (treatment-induced)

Unfortunately, despite enormous research, there are no promising treatments for PDN other than strict glycemic control and lifestyle modifications (11). A Cochrane review composed of all clinical analyses on PDN revealed that strict glycemic control in T1DM subjects can decrease the episodes of PDN, but this has little effect in T2DM subjects despite more than 10 years of glycemic control (12). This difference in incidences of PDN is extensively informative and favors the ingredient that, different pathophysiological mechanisms underlie T1DM- and T2DM-associated PDN, and it is to be considered as two diseases with similar clinical presentation (12). The pharmaceutical market has left the PDN due to a lack of basic understanding of the mechanism and research, while the enormity of the complications has reached epidemic proportions (13).

The nervous system plays a crucial role in maintaining human biological events such as cognition and individual cell function. Damage to the state of the nerve may cause tremendous consequences that may be life-threatening. Peripheral nerve degeneration has increased over the past few years affecting 2.8% of trauma subjects annually (14). PDN is likely to be associated with demyelination and axonal atrophy that leads to nerve degeneration as suggested by animal experiments (15, 16). The patho-mechanistic approach leads to damage of peripheral nerve plexus in PDN including two major components. First is the impairment of endoneurial circulation following ischemia (17) and the second is abrupt axon-glia relationships followed by segmental or paranodal demyelination (18), Wallerian degeneration and neuroma (19). Despite this conundrum, PDN-related research in the last decades has focused on the therapeutic potentials of biomaterials in PDN that directly improve the severe form of complications.

**Figure 2:**
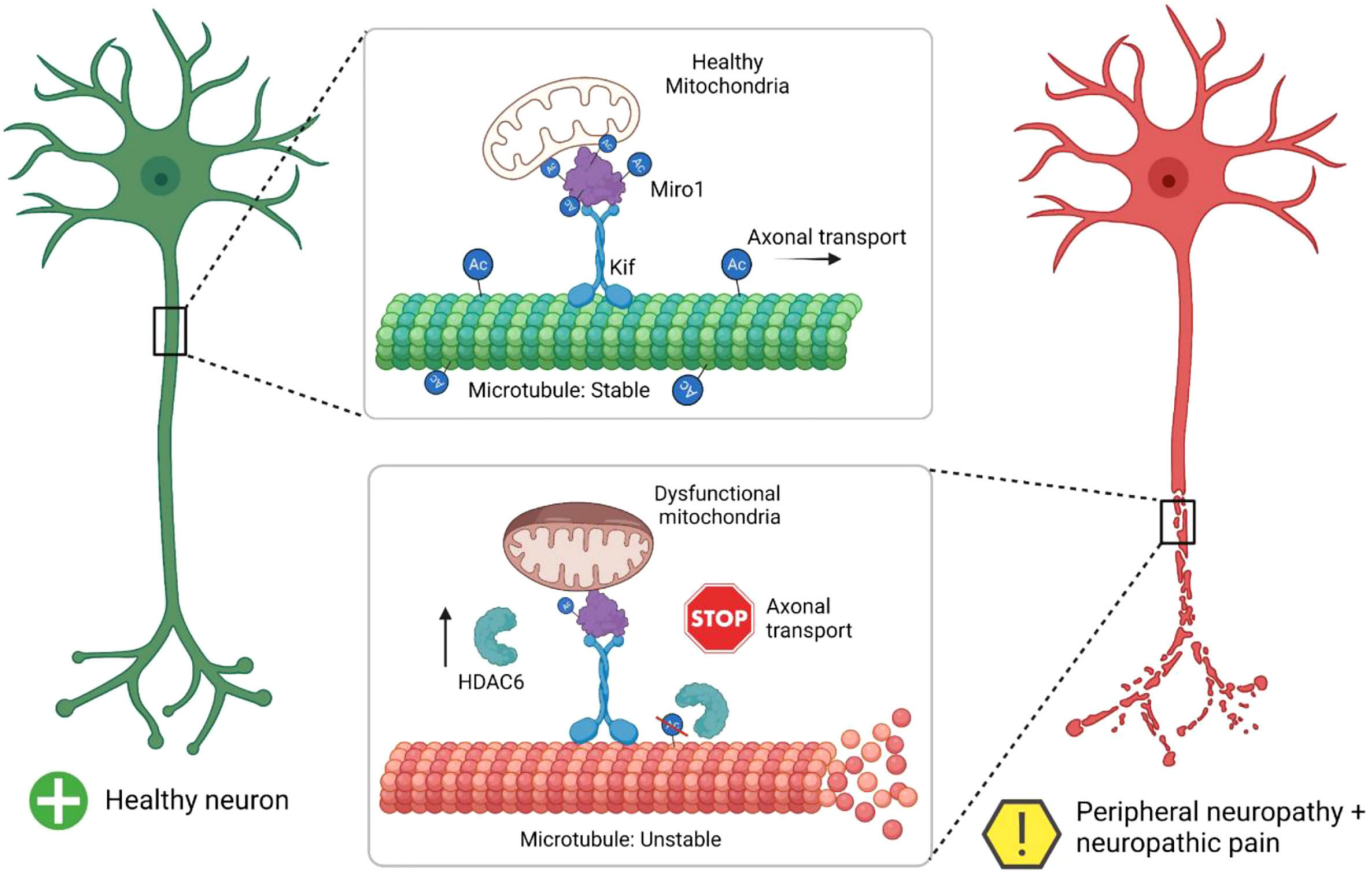
Summary of a healthy neuron undergoing neuropathy (Adopted from Ref. 20 under common creative license).

A previously published study has demonstrated the repair of peripheral nerve damage with a common goal of regenerating nerve fibres at their proper place (i.e. endoneurial tubes) and maintaining functional recovery (20). The details of the conversion of a healthy neuron to a peripheral neuropathy neuron are demonstrated in **Figure 2**. Implantation of autografts, allografts, and xenografts from donor subjects are some of the strategic approaches available at present. The few demerits associated with these grafts primarily include loss of function or sensation at the donor nerve graft site, mismatch of the damaged nerve, and the abrupt dimension of nerve graft (20, 21). Furthermore, the allogenic and xenogenic also show disease transmission and immunological complications (21).

Tissue engineering gives new hope to regenerative medicine as an alternative to basic conventional transplantation methods that help to modulate cell behavior and tissue progression with the application of novel biomaterials. In one of the previously published studies related to regenerative tissue engineering, the researchers fabricated polymeric scaffolds seeded with nerve cells or neural origin to produce a 3D functional tissue with regenerative capability (22). Tissue engineering strategies proved to be landmark efforts to provide a therapeutic role in peripheral nerve damage induced in diabetes mellitus (i.e. PDN), spinal cord injury, and drug delivery approach (23). Nerve guidance channels (NGCs) enabled biomaterials are specially designed implants for nerve repair. A specially designed conduit with NGCs having desired topography, dimensions, stability, and biodegradability is implicated in nerve regeneration (24). Previous research has successfully demonstrated that the electrical stimulation of these conduits used for nerve cell regeneration showed better results and effective cues in stimulating the proliferation and differentiation of neural cells (25).

In this review, we discuss the pathophysiological mechanism of peripheral diabetic neuropathy with up-to-date understanding. The study also outlines the economic burden of PDN on society. The most important part of this review is focused on the therapeutic potential of conducting biomaterials-enabled scaffolds or cryogels in nerve cell regeneration. A brief evaluation of electrical stimulation approaches of conductive polymers for tissue engineering is also discussed.

## Economic burden of peripheral diabetic neuropathy

Several studies published on the economic status of peripheral diabetic neuropathy showed that patients with these complications bear high costs to manage their health. One study conducted in Europe mentioned statistical data that 76% of PDN patients visited their physician at least once in 4 weeks (26). In one study, the healthcare cost of PDN patients in the UK was £2,511 of which 41% of the budget accounts for patient care (27). Drug costs involved in the treatment and management of DPN account for 30-32% of the total healthcare budget (27). Per patient, drug costs vary with the choice of effective medications. The cost of presently available generic anti-depressants is less compared to newer molecules employed in the treatment and management of PDN. In another study conducted in the UK region, it has been found that peripheral diabetic painful neuropathy (PDPN) is significantly associated with discontinuous employment and work productivity (26). It has been also reported that 35% of the patients with PDPN have some sort of disturbances in their employment due to pain associated with it (26). The situation worsens, as 59% of patients showed less productivity at a certain period of time due to PDPN (26). It is interesting to note that in this study that 14% of patients with mild pain showed work disruption, 38% of patients showed moderate pain and had an interruption to their work, and the figure was more than tripled for patients presenting with severe pain (i.e. 48%) (26).

**Figure 3:**
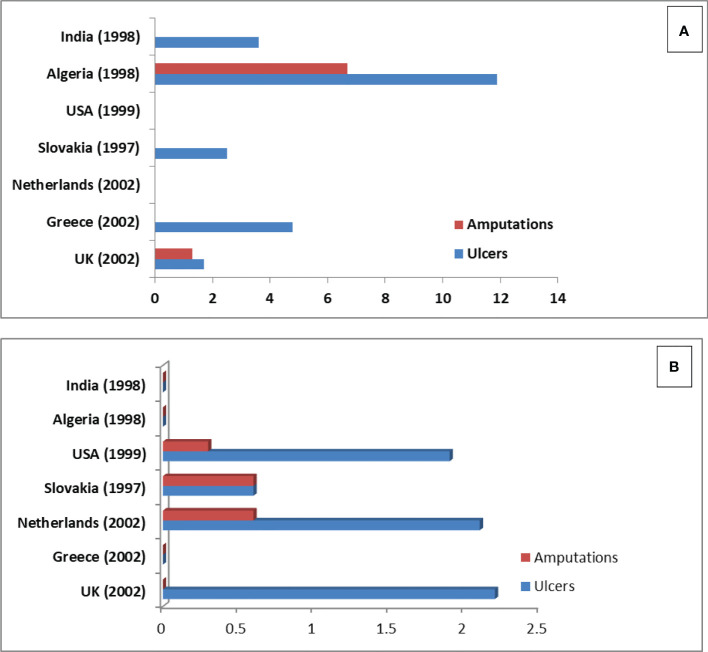
Epidemiology of foot ulceration and amputations by country **(A)** Prevalence **(B)** Incidence.

PDPN patients with microvascular complications showed the lowest EQ-5D utility value (0.63, 0.65, 0.56 *vs* 0.41) compared to macro-vascular complications of diabetes including CAD (26, 27). Similarly, other studies have shown that utilities are lower for PDPN patients compared to other chronic diseases such as depression, asthma, and Parkinson’s disease (26, 28, 29). In one of the previously published studies, Hoffman et al. (30) stated that patients presenting severe to moderate pain in PDPN had a greater mean improvement in overall health score compared to those with moderate to no/mild pain post-treatment. Data for economic burden in peripheral diabetic neuropathy was rather limited. Studies related to economic burden were done with patients in Spain (31) and the UK (26). The global burden of diabetic foot disease has been studied by Andrew et al. (2005) (32) (data shown in **Figure 3**).

## Makeover of the peripheral nervous system

The plexus of the peripheral nervous system (PNS) comprises 12 cranial nerves and 31 pairs of spinal nerves along with neurons and glial cells (especially Schwann cells). The central nervous system (CNS) coordinates with muscles and glands *via* efferent axons. Furthermore, afferent axons coordinate between CNS and peripheral sensory receptors. A long network of axons sometimes poses a unique challenge to PNS (33). Likewise, peripheral sensory receptors are sensory neurons (i.e dorsal root neurons) located outside of the blood-nerve barrier. Moreover, motor neurons are situated in the vicinity of the ventral horn of the spinal cord spanning the blood-brain barrier (BBB). Motor neurons remain protected, enabling dorsal root ganglion neurons susceptible to systemic metabolic hypoxia-induced injuries. PNS is composed of thin (<1nm) myelination-devoid axons known as C fibers. Furthermore, several unmyelinated axons, more than myelinated axons are components of PNS. Feldman et al. (2005) reported that these C fibers are termed the “foot soldiers” of PNS and were discovered by the late Jack Griffin, a pioneer scientist in the peripheral neuropathy field (34).

The PNS is also constituted of the afferent myelinated fibers that are responsible for the transfer of information from peripheral receptors to the location of sense and touch. The mechanism of myelination of these afferent fibers by Schwann cells (SCs) is typically controlled and forms differentiated nodal domains such as; the node of Ranvier, the paranodes, and the juxtaparanodes. Vast variations in the anatomy of unmyelinated and myelinated fibers occur in diabetes and its related complications. Pre-diabetes is also among the important factors for such changes. The first case report in the field of diabetic peripheral neuropathy enabled changes in PNS was published in 1890 by Auche and their coworkers that further reviewed by Colby in the year 1965 (35). Electron microscopy also has a history associated with PDN. In the 1960s, for the first time, sural nerve biopsies (anatomical studies) from patients with diabetic neuropathies (DN) were performed by P.K Thomas, P.J Dyck, and their co-workers. The previous studies, along with current reports, mentioned changes in unmyelinated C fibers in DN subjects (36). Recent literature also reported that the degeneration and regeneration of these C fibers result in pain, hyperesthesias, and allodynia (37). A study done on pre-diabetic subjects revealed that over a long period the degeneration mechanisms override the regeneration, resulting in the loss of C- fibers (38). Axonal loss occurs due to the loss of nutrient flow from SCs to myelinated axons in DN (39). To summarize this section, there is now a clear picture of PNS alteration in diabetic neuropathy. We will now discuss the biochemical pathways that are widely known to be dysregulated in PDN associated with diabetes mellitus.

## Biochemical pathways implicated in peripheral diabetic neuropathy

Over the last few decades, research in the sector of peripheral diabetic neuropathy has focused on the detailed mechanisms and biochemical pathways associated with nervous system damage. The studies done previously have focused on enhancing the pathological relevance of polyol pathway activation, hexosamine/protein kinase C (PKC) isoform activation, accumulation of advanced glycation end products (AGEs) in the peripheral nerves plexus in a diabetic state, and oxidative stress. Each specific pathway alone is injurious to the nervous network and may cause severe complications in subjects with diabetes mellitus during prolonged and uncontrolled hyperglycemia.

### (a) Polyol pathway

The polyol pathway is the most highlighted biochemical mechanism in diabetic neuropathy. Under normoglycemia, glucose undergoes glycolysis and other biochemical inlets for energy generation and storage. Moreover, during hyperglycemia, excess glucose is converted into sorbitol (alcohol) by an enzyme aldose reductase (EC No. 1.1.1.21), leading to increased sorbitol levels and osmotic imbalance. This event results in osmotic stress and compensatory efflux of myoinositol and taurine. Myoinositol is an essential component of sodium/potassium ion (Na^+^/K^+^) ATPase and therefore, its loss impairs nerve makeover and function.

Aldose reductase activity decreases the reservoir of nicotinamide adenine dinucleotide phosphate (NADPH) required for nitric oxide (NO) and essential antioxidant generation (i.e glutathione). The generation of cytoplasmic-reactive oxygen species (ROS) leads to intracellular injury and cellular dysfunction. This concept is known as the “metabolic flux” hypothesis, as reviewed by Oates and their co-workers (40). A pre-clinical rodent study performed on a streptozotocin (STZ) rat model with T1DM showed the relation between the polyol pathway and impairment in PNS makeover and function (41). Exploiting this premise, several AR inhibitors (ARI) have been developed and tested for pre-clinical aspects, but the clinical trials have shown the failure of promising results due to adverse effects at the clinical end-point of the studies. Currently, epalrestat (ONO2235) is the only licensed ARI in Japan as it was approved after the 3-month double-blind trials which showed promising improvement in nerve function (42). In another study conducted for ARI, it has been found that it ameliorates nerve conduction velocity (NCV) and nerve makeover in the diabetic state (43). The failure of ARI in treatment has been attributed to experimental design, inaccessibility to PNS, dose range, and secondary hepatotoxicity (44). The advent of recent and promising transgenic technology has revealed further insights into the polyol pathway. A study conducted on a galactose-fed transgenic mice model revealed that it overexpressed the human AR and developed severe diabetic neuropathy (45). Despite ample pre-clinical research, a detailed mechanism of the influence of the polyol pathway in neuropathy is still unclear.

### (b) Hexosamine/PKC pathway

Increased activity of the glycolysis pathway is the response to hyperglycemia and also impairs the metabolic pathways and promotes neuronal injuries. The fructose-6-phosphate is a glycolytic intermediate that enters the hexosamine pathway and follows a series of treatments to form uridine-5-diphosphate-N-acetylglucosamine (GlcNac). GlcNac has the ability to bind serine/threonine amino acids to transcription factors, particularly Sp-1, resulting in lipid dyshomeostasis and injury to peripheral nerves along with inflammation (46). Dihydroxy-acetone phosphate (DHAP), another glycolytic intermediate converted into diacyl glycerol (DAG), is responsible for the initiation of tissue complications, especially of nerves due to activation of the neuronal protein kinase C (PKC) (47).

PKC inhibitors have also been tested for promising therapeutic results in peripheral diabetic neuropathy. In one study done on STZ-induced diabetic rats, it has been found that there is an improvement in nerve physiology and function with the use of PKC inhibitors in the treatment of diabetic neuropathy (48). In one of the landmark studies done on human subjects, the PKC-β inhibitor (ruboxistaurin) was found to have an improving role in the treatment of diabetic neuropathy but unfortunately was not effective in diabetic retinopathy and nephropathy (48). PKC is a key player in the pathogenesis of diabetic neuropathy (48). In their study, Nakamura and their co-workers did not find any significant changes in PKC activity in the tissue homogenate of whole peripheral nerve tissue in a diabetic model induced by STZ, although there was an improvement in nerve conduction velocity and blood circulation to the nerve upon using PKC-β inhibitors (49). It is presumed that using PKC-β inhibitors may prove useful in the treatment of peripheral diabetic neuropathy. In a pre-clinical study performed on animal models, PKC-β inhibitors have beneficial effects on neuropathic changes in STZ-induced diabetic rats (50). Despite successful pre-clinical animal studies, the clinical trial showed failure, in part, to the high improvement rate in the placebo group (51).

### (c) Advanced glycation end-product pathways

Advanced glycation end products (AGEs) are irreversible glycation products formed *via* the Maillard reaction between glucose and amino groups of proteins. AGEs enable the cross-linking of proteins, thereby altering their structure and function. AGEs bind with the extracellular cell surface receptors, the receptors of AGEs (RAGE), which activate the nuclear factor kβ (NF- kβ) that initiate a series of complications like vasoconstriction, inflammation, and neurotrophic support loss of PNS (52). An accumulation of AGEs in the peripheral nerves was also found in patients with diabetic neuropathy in T2DM subjects (53). A previously reported study has revealed the importance of the RAGE-AGEs relationship in the pathogenesis of DN (54). Potent AGEs like di-carbonyl active species have been reported to damage sensory neurons (55).

An *in-vitro* cell line study on rat Schwann cells showed a decrease in viability in the presence of glycolaldehyde (precursor of AGEs) in a physiological environment, thus contributing to the progression of diabetic neuropathy (56). The presence of CML was reported in vascular endothelial cells, pericytes, and basement membranes of axons and Schwann cells in diabetic peripheral nerves. *In-vitro* incubation of neural cells in an AGEs-rich medium induces cell death (57). It is hypothesized that uncontrolled hyperglycemia initiates the AGEs formation and their accumulation in the peripheral nerve affects the structural and functional characteristics of proteins, thereby adversely affecting the function of the nervous system and leading to diabetic neuropathy.

There are two proposed mechanisms of diabetic neuropathy induction *via* protein AGEs. First, the advanced glycation mechanism tends to impair the biological functions and properties of the protein, thus making abnormal neuronal activity (58). Secondly, the lipid and protein AGEs bind to RAGE to initiate the signalling cascade that elevates the oxidative stress and neuronal cell injury mediated by NADP(H) oxidase (59) along with nitrosative stress (60). In one study, it has been reported that there is a structural and functional abnormality in axons, Schwann cells, and dorsal root ganglia neurons when the AGEs bind to RAGE in experimental diabetic rat and mouse models of neuropathy (61). The AGE-RAGE axis appears to mediate the prolonged cellular pro-inflammatory response in chronic diabetic complications (62). In one of the studies related to NCV, the supply of exogenous AGEs showed a delay in NCV in the diabetic neuropathy model (63). With this delay in NCV, the activity of nerve Na, and K-ATPase declined. As a result of this, the myelinated nerve fibers shrunk in size. Furthermore, preliminary clinical trials in diabetic neuropathy showed improvement with the use of benfotiamine, but there is still no molecule found that can suppress the formation of AGEs and improvement of peripheral diabetic neuropathy in humans.

### (d) Oxidative stress

Enhanced glycolytic flux in hyperglycemia leads to the generation of free radicals that are primarily responsible for diabetic neuropathy (64). Numerous studies proved that oxidative stress (OS) induces peripheral nerve injury in experimental diabetes (65–67). Using this information, the studies attempted to improve neuropathic complications with antioxidants (41, 68). A previously published study used α-lipoic acid for the suppression of OS in diabetic animal models that showed improvement in NCV delay (69). A most important event of programmed cell death occurs due to the release of cytochrome-C, and caspase-3 activation (70). Oxidative stress is defined as the imbalance in the antioxidant defense mechanism. The majority of reactive oxygen species (ROS) are the products of mitochondrial respiration that cause cellular and molecular damage. In the state of hyperglycemia and excess fatty acid flux, there is excess substrate metabolized through glycolysis and the TCA cycle, respectively, enriching the dorsal root ganglion (DRG) neurons with ample NADH and FADH_2_ electron donors. This increases the proton gradient and, hence, ROS generation. The one-electron reduction process of molecular oxygen generates a stable intermediate, known as superoxide anion ( $\text{O}\cdot_{2}^{-}$ ) that serves as a precursor of ROS. The superoxide dismutase (EC No. 1.15.1.1) performs the dismutation of the superoxide anion that produces H_2_O_2_. Furthermore, the interaction of H_2_O_2_ and $\text{O}\cdot_{2}^{-}$ in a Haber-Weiss reaction or Fenton reaction generates a highly reactive hydroxyl radical (OH). Mitochondrial damage decreases neurotrophin-3 (NT-3) and the nerve growth factor (NGF) that disturbs neurotrophic support. It is important to note that a small amount of insulin is insufficient for lowering systemic blood glucose levels and is capable of enhancing the NCV delay (71). Moreover, nitro-oxidative stress activates poly ADP-ribose polymerase (PARP) in conjunction with sustained hyperglycemia (72), resulting in cellular dysfunction and programmed cell death that can be inhibited by using PARP inhibitors (73).

### (e) Insulin physiology

Studies related to DN have evolved from the hyperglycemic viewpoint, which points out DN has a complex of secondary complications associated with metabolic and oxidative insults as described in the above sections. T1DM and T2DM exhibit different presentations of DN with different occurrence mechanisms in each disorder (74). In subjects with T1DM, DN improvement relies on different insulin shots (dose) and is suggestive of the fact that one or more insulin shots do not show strict glycemic control but have a positive impact on PNS (75). Insulin receptors (IRs) are well expressed on sensory neurons and motor neurons and showed enriched presence in SC membranes along with nodes of Ranvier. Furthermore, the role of insulin signaling is not fully understood. Insulin binding to IRs activates the signaling cascade of intracellular mechanisms that helps in maintaining normal cell homeostasis (76). It is also studied in previous literature on T1DM with DN complications that the failure of insulin signaling leads to cell injury and promotes apoptosis (77). The improvement in PNS in DN is either due to normoglycemia or insulin-mediated neuronal signaling, but this is still a matter of debate.

## How is diabetes injurious to axons?

It is speculated that SCs provide energy to axons. In the state of diabetes mellitus, the SCs not only lose their capability to provide energy to myelinated and unmyelinated axons but are also involved in the transfer of harmful lipid molecules to the point of contact of axons. The puzzle of axonal vulnerability during diabetes is difficult to decipher (78). SCs produce acylcarnitines that trigger the influx of extracellular calcium ions inside the axon which disturbs axonal mitochondrial trafficking, resulting in insufficient energy generation and mitochondrial apoptosis (78). Moreover, the diabetic rodent model showed elevated mitochondrial ROS in axons that lead to dysfunction and axonal injury (78). *In-vitro* studies on DRG neurons speculated that inhibition of mitochondrial complex 1 decreases ATP generation that in turn increased ROS generation, leading to axonal degeneration (79).

The abundant ion channel presence in axons makes them vulnerable to diabetes-associated injury. Axons are known to exhibit numerous voltage-gated sodium channels along with sodium-calcium exchanger pumps in their termini (80). Na/K ATPase pump performs an export of intra-axonal Na+ entry to generate an action potential, however, low ATP levels fail to generate this action potential and, in turn, leads to axonal degeneration. Sensory axons exhibit distinct voltage-gated sodium-calcium ion channels (Na_v_1.6, 1.7, 1.8, and 1.9) compared to motor axons (Na_v_ 1.6) with a distinct biophysical makeover (81). The sensory neuron because of its short diameter, interplays Na+/Ca++ changes rendering them to axonal injury. In conclusion, the vulnerability of axons in diabetes mellitus-induced peripheral diabetic neuropathy is secondary in combination with energy factors and ion channel presentations.

The above sections give a focused message that there is severe to mild injury associated with the peripheral nervous system in diabetes-associated PDN. The goal of future research should be to provide a therapeutic approach for the management of neuronal injury associated with PDN. The advent of biomaterials shows promising potential for the cure of nerve injuries in PDN. Biomaterials with the appropriate property of conduction and nerve homology provide the prospects to provide relief to mankind’s society. Our lab is still trying to fulfill this requirement around the clock. The next section of this review deals with biomaterials and their role in PDN.

## Introduction to biomaterials

In general, biomaterials are defined as “any natural or synthetic material comprise of living component, which is capable of replacing and performing the function of essential tissue where it has been placed”. Park and Lakes state that biomaterial is any synthetic material capable of replacing a part of a living system in every respect. Clemson University Advisory Board for Biomaterials state that “it is synthetically and pharmacologically inert material with a motto of implantation into the living system”. Williams, in 1987, proposed that biomaterial is an implanted source intended to couple with the living system (82). Regarding tissue engineering, biomaterial serves the purpose of repairing and reconstructing the damaged and injured part of a biological system. A few examples where biomaterials can be used as a substitute include heart valves, artificial hips, dental implants, and fixing fractures. So, it can be concluded that any material with specific properties of regeneration can fit into the criteria of biomaterials. It is also stated in the previously published literature that any material with the property of biocompatibility can be considered biomaterial (83). Biomaterials can also be used to deliver molecules or essential factors required for the regeneration of tissue (84).

## How many choices are there for biomaterials?

Biomaterials, as discussed in the above section, have a regeneration ability with premium characteristics of bio-compatibility and similar tissue physiology. The biomaterials are broadly grouped into three choices:

### Polymers

Polymeric biomaterials perform other functions apart from tissue regeneration such as fixing fractures with bone cementing materials (e.g. poly methyl methacrylate), or polyglycolic acid being used in degradable surgical sutures. Polymers in tissue engineering can be both synthetic and natural. Polymers consist of repeating units of monomers. There are a variety of synthetic polymers used in regenerative engineering and medicine. These include silicone rubber (SR), polypropylene (PP), polyethylene (PE), poly (ethylene terephthalate) (PET), polyvinyl chloride (PVC), polytetrafluoroethylene (PTFE), and polyethylene glycol (PEG), along with others not included in the list. These polymers have a role in regenerative medicine and tissue engineering. Polymers with mucoadhesive properties can be used for mucosal drug delivery, while polyacrylics are used for dental implants. PP because of its good tensile strength can be used for degradable sutures. Moreover, poly lactic-co-glycolic acid (PLGA), a Food and Drug Administration (FDA) approved biodegradable polymer, is used in resorbable surgical sutures and drug delivery and orthopedic applications as a fixative.

Natural polymers also have applications in regenerative tissue engineering and include alginate, collagen, fibrin gels, hyaluronic acid, etc (85). Alginate biopolymer contains repeating monosaccharide units of i.e L-guluronic acid and D-mannuronic acid that are useful in the generation of three-dimensional gel. Alginate can be used for cartilage regeneration as alginate gels. Another natural polymer, collagen, has been implicated in skin regeneration, as it is an essential component of human skin. A previously published study used bilayered collagen to regenerate skin (86). Hyaluronan, also a natural polymer with certain modifications, can be used as a degradable scaffold in tissue regeneration and can be used for cartilage repair (87). Fibrin is another natural polymer derived from animal skin and is applicable for wound healing. This property can be exploited as a scaffold with the disadvantage of frequent fibrinolysis and fast degradation. To overcome this problem, a slight modification has been employed, so it can be used for a longer period (88).

### Metals

Biomaterials can be used in the form of metals apart from synthetic and natural polymers. Metals can also serve a major role in tissue engineering and have a significant economic impact on tissue regeneration and biomedicine. Metallic implants like steel 316, 316L, cobalt, F75, Vitallium, silver, tantalum, and alloys of Ti, Cr+, Co, Mo, etc., can be successfully used for implants in tissue engineering. Metallic implants have associated disadvantages such as corrosion susceptibility and low biocompatibility (89). Metallic implants can be used in tooth implants, penis implants, and in facial reconstruction (90).

### Ceramics

Ceramics is another category of biomaterials used in tissue engineering. Ceramics are broadly used as a component of eyeglasses, diagnostic instruments, thermometers, etc. These classes of biomaterials possess high biocompatibility, high resistance, and reduced thermal and electrical conductivity, which makes them suitable for implants (91). New bone tissue regeneration uses hydroxyapatite (HAp), a class of ceramics. Other ceramics include aluminum oxide, titanium oxides, calcium phosphate, calcium aluminates, carbon bioglass, etc. Ceramics exhibits disadvantages of low impact resistance and difficulty in fabrication. The major achievements with the use of ceramics include heart valve implants, hip implants, and knee implants as demonstrated by landmark studies in tissue engineering (92, 93). The elaborated list is depicted in **Table 2**.

**Table 2:** State-of-the-art materials for long-term implants.

Materials	Applications
Titanium alloys	Dental implants, femoral stems, pacemaker cans, heart valves, fracture plates, spinal cages
Cobalt–chromium alloys	Bearing surfaces, heart valves, stents, pacemaker leads
Platinum group alloys	Electrodes
Nitinol	Shape memory applications
Stainless steel	Stents, orthopedic implants
Alumina	Bearing surfaces
Calcium phosphates	Bioactive surfaces, bone substitutes
Carbon	Heart valves
UHMW polyethylene	Bearing surfaces
PEEK	Spinal cages
PMMA	Bone cement, intraocular lenses
Silicones	Soft tissue augmentation, insulating leads, ophthalmological devices
Polyurethane	Pacemaker lead insulation
Expanded PTFE	Vascular grafts, heart valves
Polyester textile	Vascular grafts, heart valves
Cryogels	Nerve Implants

## Characteristics of biomaterials enabling its clinical application

The first and foremost characteristic of biomaterials is biocompatibility both *in-vitro* and *in-vivo*. *In-vitro* evaluation of biomaterial with an *in-vitro* cell setup is an inexpensive primary approach for testing its interaction. Moreover, the results of the *in-vitro* testing are not useful until the desired biomaterial is not able to fulfill the *in-vivo* implant motto. Cytotoxicity assessment (i.e. apoptosis, alterations in cell permeability) of biomaterials for the determination of biocompatibility is a primary test for its implant viability in regenerative medicine. Biomaterials that initiate cell apoptosis (death) or changes in cell properties may be proved toxic and are not suitable to use as an implant. If the biomaterial passed the cytotoxicity examination, then it has to qualify for the next test of biocompatibility performed on a popularly used mammalian fibroblast cell line (L-929 cells) and MTT (3­(4,5­dimethylthiazol­2­yl)­2,5­diphenyltetrazolium bromide) assay. Experiments on a suitable animal model system are the final step for testing the regenerative ability of the biomaterials in implants (**Table 3**). The biomaterial implants with desired characteristics are placed at the site of damage or defect, and further experiments on animals are performed to prove their biocompatibility and ability to regenerate. Moreover, post-implantation, the hemocompatibility and biodegradation of the implant are tested. The combo of both *in-vitro* and *in-vivo* testing is a necessary part of tissue regenerative engineering. Currently, the reforming of a damaged nerve is conducted using nerve autografts. Such treatment modalities can be replaced with engineered conduits suitable for nerve lesions and large nerve defects. Carbon nanotubes (CNTs) are considered to be a novel biomaterial with vast biomedical applications. CNTs exhibit unique characteristics that could be used for nerve repair in the form of conduits. CNTs have a cylindrical shape with a nanosized diameter along with properties like flexibility, electrical conductivity, and biocompatibility.

**Table 3:** Animal model for *in-vivo* implants experiments.

Implant application	Animal model system
Cardiovascular	
(a) Heart Valves	Sheep
(b) Vascular graft	Dog, Pig
(c) Artificial Heart	Calf
Orthopedic/Bone	
(a) Bone regeneration	Rabbit, dog, pig, mouse, rat
(b) Total hip/knee joint replacements	Dog, goat
(c) Vertebral implants	Sheep, goat, baboon
Neurological implants	
(a) Peripheral nerve regeneration	Rat, cat, non-human primates
(b) Electric stimulation	Rat, cat, non-human primates
Ophthalmological	
(a) Contact lens	Rabbit
(b) Intraocular lens	Rabbit, monkey

## Unique classes of scaffolds: A boon for tissue engineering

The conventional biomaterials discussed in the previous section can be used as implants after fabricating into three-dimensional scaffolds to achieve the desired functions of growth, proliferation, and regeneration in tissue engineering. The above biomaterials can be fabricated into desired implanting scaffolds with varied fabrication technologies such as (i) solvent casting mechanism in combination with leaching, (ii) fiber building network, (iii) phase separation with freeze drying, and (iv) solid free form fabrication (94). Furthermore, with the advancement in technologies, the advent of next-generation biomaterials for tissue regeneration and repair can serve the regeneration purpose well. These next-generation implant biomaterials not only regenerate the desired tissue but also serve as factor-delivery vehicles that provide nourishment and an essential growth environment to the tissue. Below are the next-generation biomaterials that can be used as scaffold implants successfully.

### Next-generation hydrogels

Hydrogels are the class of colloidal gel in which water is used as the dispersion medium. These biomaterials are used as implant scaffolds in tissue engineering, as they resemble the topography and function of native tissue. The aqueous behavior of these gels enables them to resemble the cells in the body. Moreover, due to the presence of fine porosity, nutrients and waste factors are exchanged easily. Natural and synthetic biopolymers can be used in tissue regeneration after the fabrication of these biopolymers into hydrogels (95). Synthetic hydrogels implemented in tissue regeneration include (PU), polyethylene oxide (PEO), poly (N­ isopropyl acrylamide) (PNIPAAm), polyvinyl alcohol (PVA), polyacrylic acid (PAA), and poly propylene furmarate­co­ethylene glycol [P(PF­co­EG)], while the natural hydrogels include agarose, alginate, chitosan, collagen, fibrin, gelatin, and hyaluronic acid (96).

### Next-generation cryogels

Cryogel application is a landmark achievement in the field of tissue regeneration. Cryogels are supermacroporous gel matrices formatted in mold-freezing conditions. Cryogels are the gel matrices prepared by crosslinking and polymerization, at sub-zero temperatures, from either a synthetic or natural source of biopolymers without involving any inorganic solvents. Cryogelation enables a scaffold to be formatted into diverse forms like disks, sheets, and monoliths with desired dimensions due to the presence of large and interconnected pores that provide several advantages over the conventional scaffold. In the cryogelation technique, there are two parts to the solvent; one part of the solvent used remains unfrozen (liquid microphase), while the other part of the solvent remains in the frozen state. This two-phase mechanism of solvent provides a platform for monomers and polymers for chemical reactions. The unfrozen liquid microphase is the best candidate for gel formation and further undergoes chemical modifications to form a porous scaffold, also known as porogens, upon thawing. This macroporous scaffold provides an undisturbed and continuous supply of growth factors, nutrients, gases, and liquids to the regenerative tissue (97–99). These cryogelation synthesized scaffolds provide an interconnected pore (200 mm) for the supply of essential factors needed for cell proliferation.

Cryogels can be synthesized from either monomeric or polymeric precursors that are uniformly dissolved in a solvent system and later subjected to a frozen environment for cryoconcentration of these precursors in an unfrozen liquid phase (100). Water is the best choice in the list of solvents due to its biocompatibility and environmental friendliness. Moreover, the application of water limits the choice to water-soluble compounds only, thus resulting in hydrophilic cryogels. It is also suggested by previously published studies that hydrophilic cryogels can be prepared by using organic solvents such as dimethylsulphoxide, dioxane, nitrobenzene, formamide, benzene, and cyclohexane (101–103). The cryogels synthesis can be performed by: (i) covalent chemical cross-linking of polymeric materials and free radicals polymerization; and (ii) non-covalently by cryogelation method, through the non-covalent structure and entrapment.

## Gels and cryogels

The gels referred to as polymeric macromolecules are immobilized and form a three-dimensional network with non-fluctuating bonds. The method of gel preparation and the nature of the bonds contribute to the morphology of the gel creation along with the chemical structure of the polymers. Solvent immobilization within a three-dimensional polymer system plays an important role as it does not allow the polymer to be compact, and hence avoids the collapse of the gel system. Depending upon the nature of the intermolecular network of bonds, the gels can be classified into two categories; (i) chemical gels and (ii) physical gels (details not included). Once the chemically reactive groups in the gel matrices are exhausted, the reaction halts, leaving behind the solid gel with interconnected polymeric materials. Considering the biomedical application of cryogels, they have to be considered a candidate of increasing concern. The cryogels are formed by the cryotropic gelation process that produces the polymeric materials with the desired morphology when compared to non-frozen gels. Cryogels are formed at a sub-zero temperature that allows the freezing of solvent molecules (free from the solute part). Ice formation in water occurs when it is exposed to below-freezing temperatures. During the formation of ice crystals, only the water part remains in the ice lattice, expelling all solutes out of the crystals (104). The formed ice crystal acts as porogen leading to the formation of a supermacroporous cryogel (105). Cryogels could be of covalent, ionic, or non-covalent types. In the cryogel system, solvent freezing followed by the sublimation of solvent crystals (in an aqueous system), forms an interconnected porous network of polymeric biomaterials. Moreover, no gel formation occurs in the unfrozen microphase. The cryogels can be modified according to the desired characteristics and their use.

## Statistical trend of cryogel research

This section demonstrates the current state of cryogels-related research and its statistical perspectives. Initially, cryogels were studied by a few research groups, but in the latter part of the 20^th^ century, the advancement in cryogel applications made them famous, and more research groups were attracted to them. In the beginning, cryogels applications rose exponentially due to their wide suitability in the biomedical field. As time passes, cryogels have attracted more research groups to explore their characteristics for the welfare of human mankind. More than 40% of cryogels-based research describes in detail that it can be used in separation and molecular imprinting due to its macroporous behavior. This especial feature of cryogels attracted more research groups (106, 107).

## Application of cryogels in the neural regeneration domain

Macroporous materials have attracted a lot of attention and also have been employed in wide applications in the field of biomedicine and biotechnology. A large pore size of 10-200µm, makes a cryogel suitable for transporting red blood cells (RBCs) of ~7 μm *via* liquid flow through a monolithic cryogel column (108). Human acute myeloid leukemia KG-1 cells expressing the CD34 possessing surface antigen and human blood lymphocyte fractions were assessed by using polyvinyl alcohol beads and dimethylacetamide (DMAAm) monolithic cryogel columns immobilized on protein A. This setup showed approximately 76% retention of cells (108). Furthermore, the application of cryogels in biomedical and biotechnology has been discussed in detail by previously published studies (108, 109). This review is especially focused on the use of cryogels in nerve cell regeneration and the management of peripheral diabetic neuropathy. Treatment of injured neuronal components in the peripheral nervous system needs new medical therapies. The regenerative ability of PNS can be exploited with cryogels to successfully treat nerve injuries or damage. Current strategies use nerve autograft, but donor site morbidity or topographical anomalies (compatibility with the damaged part) do not provide a promising solution for the repair mechanism. Alternatively, the use of nerve guidance conduits (NGCs) associated with the cryogels system provides a successful solution to promote nerve repair.

Synthetic NGCs become the superior choice for surgeons to repair injured nerves instead of traditional methods of autografts, allografts, and xenografts (110, 111). NGCs are biomaterial-based systems used for nerve repair. An absolute synthetic material may be readily formed for synthesizing NGCs containing cryogels with desired dimensions and properties. The physical and chemical make-over of the cryogel affects the outcome of nerve regeneration and repair. Neurorrhaphy alone is not suitable for recovery of normal sensory and motor functions despite modern aids in microsurgical techniques. Previously published literature showed that only 50% of patients regain useful normal function post neurorrhaphy (112). Furthermore, additional additive factors are required for normal recovery in these cases. Cryogel conduits were implemented in previous studies to regenerate the peripheral nerve and supporting glial cells (113, 114). A wide range of natural and synthetic ingredients such as PLA (115), chitosan (116), and gelatin (117) have been implicated in the synthesis of cryogel nerve conduits. One of the studies conducted by Ju-Ying Chang et al. successfully demonstrated the role of the RDC/NHS-fixed gelatin nerve conduit in filling a large gap in the sciatic nerve of a rat model (117). Designing a 3D printed nerve conduit capable of delivering the essential nerve growth factors (NGFs) would serve as a promising approach for promoting the function of the sciatic nerve followed by post-end-to-end neurorrhaphy (118).

## Improving make-over of next-generation cryogels in nerve regeneration

Advancement in technology improves the make-over of the cryogel. It updated cryogels with modern properties to serve better fulfilling its goal. Research strategies for improving nerve repair can be placed in two broad categories: (i) a methodology to improve axonal regeneration and (ii) a methodology to decrease immune responses (i.e inflammation). Furthermore, methods for enhancing the axonal regeneration include (a) axonal sprouting from the distal nerve stump (electrical stimulation and growth factors of proximal stump); (b) permissible environment for exchangers and nutrients essential for growth (enhanced cryogels pores, surface topography); (c) delaying of Wallerian degeneration and (d) shortening of period for denervation of muscles.

Nerve growth factors (NGFs) are spontaneously naturally releasing components during the process of nerve regeneration. NGFs release from nerve endings if a nerve has the effect of altering growth, differentiation, and surveillance (119). NGFs are present in a low concentration in healthy nerves, but during injury, their concentration rises in the distal stump of the damaged nerve and plays a vital role in the survival of the sensory neurons (119). Moreover, other factors for nerve regeneration include the Glial growth factor (GGF), glial cell-derived neurotrophic factor (GDNF), fibroblast growth factors (FGF), neurotrophin 3 (NT-3), and others (119). The factors are fixed with the cryogel conduit system to repair nerve gap injury (1-4cm). The delivery of these factors has been shown to hasten the recovery of injured nerves compared to a conduit alone without these factors (119). However, a previous study also reported a superior outcome in nerve autograft compared to NGFs seeded conduits (120). The future use of NGFs with cryogel having sustained release and other functional characteristics may be targeted towards nerve regeneration after injury or in PDN.

The prime role of the nerve is to transfer electrical signals (conduction) for creating coordination between the brain and the body. In this area, it is necessary to impart proper nerve stimulation to the injured nerve to restore the function of electrical conductivity. Limited research has been done on the implementation of the electrical fields/gradients across the peripheral nerve system for accelerating axonal regeneration. Studies performed on animals showed that post-repair electrical stimulation for one hour continuously promotes the function of target muscle reinnervation (121). Moreover, a clinical trial showed that a continuous one-hour electrical stimulation accelerates axonal regeneration after median nerve decompression in patients with tunnel syndrome and thenar atrophy (122). Conducting cryogels provides a promising tool for nerve regeneration during injury and PDN. Another study published on conducting cryogel scaffolds showed enhanced proliferation and growth of excitable cells like neuro 2a and C2C12 cells when stimulated at 100mV for 2h (123).

Besides the electrical conduction and neurotrophic arrangement, the topography also contributes to nerve regeneration. To improve the biological performance of the cryogels, numerous additive features have to be added to cryogels like topographical guidance, neurotrophic activity, and electrical activity. A previously published study showed that topography plays an essential role in nerve regeneration and repair (124).

The polymers chosen have already shown potential in the field of neural regeneration when used for 3-D scaffold synthesis in various techniques. In improving cryogels for nerve regeneration, molecular imprinting in the 3D domain imparts promising results. Molecular imprinted polymeric cryogels (MIPC) are tailor-made to offer high molecular recognition with satisfactory performances compared to non-imprinted entities. The synthesis of molecularly imprinted polymers can be done using a template-directed radical polymerization reaction. The target molecule itself acts as a molecular template, forming molecular imprints. The print molecule formed is mixed thoroughly with some functional monomers, followed by the addition of further monomers and crosslinkers preceding the polymerization reaction. The post-polymerization reaction yields the imprinted molecule after cumbersome extraction. The only associated disadvantage with molecularly imprinted polymers is compact and tight polymeric material with low pore size. Thus, a composite MIPC offers an attractive package for tissue engineering. Denizli and their co-workers published literature on composite cryogel with embedded beads of poly hydroxyethyl methacrylate formatted MIPC (125). MIPC showed the binding of human serum albumin (HSA) more efficiently following the Langmuir isotherm (125). Another reputable study showed that 3D printing technology in the synthesis of cryogels conduits for nerve regeneration showed promising results and may be used for clinical purposes (126). Another showed the role of 3D-printed biodegradable conduits in neurorrhaphy (127). The whole section discussed above concluded that cryogel with some additive features may serve a better role in nerve regeneration.

## Current therapeutic approaches to managing PDN

Prevention and management of peripheral diabetic neuropathies focus on strict glucose control and lifestyle modifications. The extensive physical, psychological, and economic burden of diabetic neuropathies underscores the urgency of casual therapies. Below are the current strategies to manage neuropathies. The management of PDN includes conventional (metabolic control and life style modifications) and recently developed methods (cytoprotective therapies and stem cell therapies).

### Metabolic control

Strict glucose control in subjects with type 1 diabetes dramatically reduces the episodes of distal symmetric polyneuropathy (DSPN) by 78% (relative risk reduction) (128, 129). Contrastingly, there is a 5-9% relative risk reduction of DSPN in type 2 diabetes (130, 131). In one small trial performed on a Japanese population with type 2 diabetes, it was found that intensive insulin treatment was related to the improvement in DSPN (132). An Action to Control Cardiovascular Risk in Diabetes (ACCORD) trial on type 2 diabetes subjects also showed a mild reduction in DSPN episodes (131). The specific glucose control approach also had some discrepancies associated with it. For example, in a Bypass Angioplasty Revascularization Investigation in Type 2 Diabetes (BARI 2D) trial male patients treated with insulin sensitizers had lower episodes of DSPN over 4 years compared to those who received sulfonylurea/insulin (133). Although, there are no randomized controlled trials of intensive insulin therapy in PDN management. Moreover, the data available showed that strict glycemic control is of greatest use in the management of PDN.

### Lifestyle modification

The appropriate model for the management of PDN includes intensive lifestyle interventions as suggested by American Diabetes Association guidelines (11). The programs launched for lifestyle interventions are the Diabetes Prevention Program (DPP), the Steno-2 Study (27), the Italian supervised treadmill study, and the University of Utah type 2 diabetes study (11). All these studies suggested that lifestyle modification can reduce the incidences of PDN/DSPN. In a previously published study, it is suggested that nerve fiber regeneration occurs prominently in Type 2 diabetes mellitus patients engaged in exercise (11). This conventional approach has had a large effect on the reduction of DSPN incidences.

## Cytoprotective therapies

### a. Lowering cell apoptosis

There is debate on whether a neuronal loss in PDN occurs. If it is happening, then there is debate whether the cells are undergoing death/apoptosis, nonapoptotic programmed cell death, or necrosis. Previously, in-vitro studies performed on rodents and cell culture models of developed neuropathy demonstrated the phenomenon of apoptosis (134–138). Mitochondria play an important role in neuronal injury that results in the depletion of dorsal root ganglion neurons (139). In diabetic neuropathy models, the activation of caspase 3, a component of the apoptotic pathway, contributes to this mechanism (140)

### b. Poly ADP-ribose polymerase inhibition

Oxidative DNA damage induces the release of a PARP factor. This enzyme is involved in DNA repair by the addition of poly (ADP-ribose) subunits to DNA strand breaks and base-excision pathway (140). It is evident from previous studies that the inhibition of PARP may delay the progression of diabetic neuropathy by inhibiting the PARP-mediated depletion of NAD+ and ATP (141, 142).

### c. Mesenchymal stem cells therapies

Current therapies for improving glucose control include the exogenous insulin supplement, which often produces the peaks of hypoglycemic episodes, as well as pancreatic islet replacement (143). The first pancreatic islet transplantation results were promising in that they decreased the long-term side effects and imparted better glycemic control (144). However, the two limitations that exist with this method for inhibiting the use in clinical trials are (i) the high demand for pancreatic islets for effective transplantation and (ii) the short-term life of transplanted islets (144). The study also stated that at least 12,000-16000 islets/Kg are required for achieving good glycemic control in each patient, suggesting the use of more than one cadaveric donor (144). The use of immune-suppressive drugs prolonged the life of transplanted islets and their associated severe complications (145). In one of the published studies, the outcome of pancreatic islet transplantation can be improved by using Mesenchymal Stem Cells (MSCs) thus limiting transplant rejection (146). Moreover, MSCs have the potential to increase cell survival, including neurons, and also to release trophic factors essential for nerve regeneration (147, 148). In one of the studies, it is shown that MSCs, along with pancreatic islet transplantation, improve glycemic control and neuropathic symptoms such as NCV. This might be due to the release of trophic angiogenetic factors (149).

## Cryogels in diabetic peripheral neuropathies: A future hope

There has been a breakthrough expansion in our knowledge and understanding of potential pathophysiological mechanisms, highlighting the neuropathies associated with diabetes mellitus that span the complete somatosensory peripheral nervous system initiating from afferent terminals to the sensory cortex. This has to be translated into a clear phenomenon-based approach for the treatment and management of PDN. The conventional approaches proved an effective system to manage PDN but have certain limitations. Moreover, experimental approaches developed recently also possess the potential to treat PDN but might also be associated with certain demerits, as discussed above. To overcome these limitations, a next-generation cryogel system must evolve with the potential for the most effective management and treatment of PDN. These may provide hope for regenerative medicine and tissue engineering. The advantage of cryogels use in various fields has already been discussed. Due to their vast application, these may be easily implemented in the treatment and management of peripheral diabetic neuropathy. The conducting cryogels can especially be used in the regeneration of damaged nerves due to injury. Peripheral diabetic neuropathy (PDN) is associated with nerve injuries or the altered behavior of peripheral nerves. Currently, conducting devices, including pacemakers, electrodes for stimulating the brain, stimulators of the spinal cord, etc. are in commercial use (150). In a previous study, it is demonstrated that polypyrrole-based cryogels scaffolds (conduits) implanted subcutaneously in rats reveal less immune cell infiltration as compared to FDA-approved poly lactic-co-glycolic acid. In another study, no inflammatory response was observed in polypyrrole-based tissue implantation (151). Similarly, polyaniline-based tissue implantation showed a low level of inflammation (151). The conducting-cryogel-based scaffold loaded with desired growth factors may prove an excellent candidate for nerve regeneration damage in PDN. Diabetic foot ulcer-initiated wounds (DFU), a severe complication associated with PDN, can be effectively solved (152–155). In summary, given the variety of mechanisms, which may overlap, a prime future goal is to better the understanding of conducting cryogels with a 3D system in clinical trials. There is now tentative evidence in the literature that supports the benefits of 3D-printed conducting cryogels (121). Moreover, these conducting cryogels may be changed according to the desired environment to treat the PDN. Conducting cryogels need to be tested in clinical trials, the outcome may be the eradication and prevention of PDN.

## Conclusion

In summary, PDN research has evolved to study deregulated pathways such as the polyol pathway, PKC, AGEs, and oxidative stress to herald a new era of insulin physiology. The disease is increasingly prevalent due to the absence of effective treatment. Biomaterials can be implemented with desired characteristics to overcome the problem of nerve regeneration in PDN and injury. Cryogel is a priority candidate in the management of PDN. Successful implantations with cryogel scaffolds loaded with growth factors and suitable topography may prove a landmark approach for the treatment of PDN beyond other conventional approaches used. The next-generation cryogel will be an effective approach to this complication.

## Author contributions

Conceptualization, AR, G-BJ, MS, RG and SA. Methodology, AR and G-BJ. Software, AR. Validation, G-BJ, SA, MS, RG, SA and SK. Formal analysis, G-BJ, SA, MS, RG and SK. Investigation, AR. Resources, SK. Data curation, AR and MS. Writing—original draft preparation, AR. Writing—review and editing, MS, RG, SA and G-BJ. Visualization, SA, MS, RG and SK. Supervision, SA, MS, RG, G-BJ and SK. project Administration, SK. All data were generated in-house, and no paper mill was used. All authors contributed to the article and approved the submitted version.

## Acknowledgments

The authors would like to thank the Multidisciplinary Research Unit, Department of Health Research, Ministry of Health and Family Welfare, New Delhi for providing financial assistance in the form of salary to AR.

## Conflict of interest

The authors declare that the research was conducted in the absence of any commercial or financial relationships that could be construed as a potential conflict of interest.

## Publisher’s note

All claims expressed in this article are solely those of the authors and do not necessarily represent those of their affiliated organizations, or those of the publisher, the editors and the reviewers. Any product that may be evaluated in this article, or claim that may be made by its manufacturer, is not guaranteed or endorsed by the publisher.
